# In this Issue

**Published:** 2011

## THE RISKS ASSOCIATED WITH ALCOHOL USE AND ALCOHOLISM

Alcohol has been shown to be an underlying cause of more than 30 diseases as well as unintentional and intentional injury, making it a major contributor to the global burden of disease. This article by Dr. Jürgen Rehm gives an overview of the disease and injury conditions that are caused by alcohol use or for which alcohol use is a contributing factor. The author also explores how analyses of these risks have influenced the development of guidelines for drinking limits. (pp. 135–143)

## DEFINING RISK DRINKING

The most challenging aspect of defining risk drinking is determining what constitutes low risk versus high risk. This article by Dr. Deborah A. Dawson discusses the conceptual and methodological challenges to defining risk drinking. She summarizes recent evidence regarding associations of various aspects of alcohol consumption with chronic and acute alcohol-related harms, including mortality, morbidity, injury, and alcohol use disorders, and discusses the study designs most appropriate to defining risk thresholds for these types of harm. (pp. 144–156)

## SCHOOL-BASED PROGRAMS TO PREVENT AND REDUCE ALCOHOL USE AMONG YOUTH

The onset of alcohol use typically begins in adolescence, making schools an important setting for the implementation of prevention programs. In this article, Drs. Melissa H. Stigler, Emily Neusel, and Cheryl L. Perry discuss characteristics of school-based prevention programs and review examples of programs that have shown promising results at the elementary-, middle-, and high-school level. (pp. 157–162)

## A REVIEW OF ENVIRONMENTAL-BASED COMMUNITY INTERVENTIONS

According to Drs. Traci L. Toomey and Kathleen M. Lenk, prevention interventions that focus solely on individual-level risk factors generally do not affect community-level outcomes and need to be reinforced by changes in the broader environment in order to achieve sustained population-level effects. The authors summarize the characteristics of community-level interventions and review evidence that such measures can help reduce alcohol use and related problems among both youth and adults. (pp. 163–166)

## ENGAGING COMMUNITIES TO PREVENT UNDERAGE DRINKING

Apromising strategy for reducing underage drinking centers on community-based efforts. In this article, Drs. Abigail A. Fagan, J. David Hawkins, and Richard F. Catalano identify several features that characterize effective community-based strategies, including reliance on local coalitions of different stakeholders; inclusion of a universal, school-based prevention curriculum; and integration of environmental strategies to change local laws, norms, and policies regarding alcohol access and use. The authors also comment on the challenges facing community mobilization efforts. (pp. 167–174)

## PREVENTION INTERVENTIONS OF ALCOHOL PROBLEMS IN THE WORKPLACE: A REVIEW AND GUIDING FRAMEWORK

Alcohol significantly affects worker productivity, and a recent national survey of 2,805 employed adults indicated that work-related impairment directly affects an estimated 15 percent of the U.S. workforce. The estimated costs of alcohol abuse were $184.6 billion in 1998, and the figures for 2010 are expected to be double that. Workplace interventions offer the potential benefit of reaching large groups of people in the settings in which they spend most of their time. This article by Drs. Genevieve M. Ames and Joel B. Bennett reviews methods of workplace prevention and discusses the most promising strategies and guidelines for combating workers’ drinking. (pp. 175–187)

## TRANSLATING FAMILY-FOCUSED PREVENTION SCIENCE INTO PUBLIC HEALTH IMPACT: ILLUSTRATIONS FROM PARTNERSHIP-BASED RESEARCH

Family-focused preventive interventions for children and adolescents have shown promise in reducing the rate of underage drinking, report Drs. Richard L. Spoth, Lisa M. Schainker, and Susanne Hiller-Sturmhöefel. To reap the full benefits of these interventions, however, the findings from family-focused preventive intervention science must be translated into real-world, community practices. According to the authors, community–university partnerships and partnership networks are one approach to help ensure such effective translation. (pp. 188–203)

## ENVIRONMENTAL APPROACHES TO PREVENTION IN COLLEGE SETTINGS

Numerous prevention interventions have been developed to combat the problem of alcohol on campus and its consequences. These interventions include individual-level interventions, community-level strategies, as well as other measures. In this article, Dr. Robert F. Saltz discusses the effectiveness of community-level programs such as A Matter of Degree, the Southwest DUI Enforcement Project, Neighborhoods Engaging With Students, the Study to Prevent Alcohol-Related Consequences, and Safer California Universities. (pp. 204–209)

## INDIVIDUAL-FOCUSED APPROACHES TO THE PREVENTION OF COLLEGE STUDENT DRINKING

Many young adults, particularly college students, use or misuse alcohol, with potentially life-long harmful consequences. Accordingly, a plethora of preventive interventions aimed at college students has been developed using a variety of approaches, including brief motivational interventions, personalized feedback interventions, personalized normative feedback, and combinations thereof. Drs. Jessica M. Cronce and Mary E. Larimer summarize the findings of several reviews of individual-focused preventive interventions in the college student population. (pp. 210–221)

## COLLEGE PREVENTION: A VIEW OF PRESENT (AND FUTURE) WEB-BASED APPROACHES

A variety of Web-based, alcohol-related programs aimed at college students are now available and being used throughout campuses across the United States. In this sidebar, Drs. Scott T. Walters and Clayton Neighbors provide an overview of current Web-based intervention strategies, their advantages and disadvantages, and their impact on college-aged youth. (pp. 222–224)

## PREVENTING IMPAIRED DRIVING: OPPORTUNITIES AND PROBLEMS

Impaired driving is a major problem in the United States, and although rates have gone down since all 50 States implemented impaired-driving laws, progress has stagnated in recent years. This halt in the decline in impaired driving can be attributed to the fact that evidence-based laws and current best practices in enforcement of these laws are not being fully adopted or used. Dr. Robert B. Voas and Mr. James C. Fell discuss several studies focused on impaired-driving intervention strategies and the need to modify laws and policies to ensure that they are implemented and enforced to their fullest extent. (pp. 225–235)

## THE EFFECTS OF PRICES ON ALCOHOL USE AND ITS CONSEQUENCES

The level of taxes on alcoholic beverages can impact alcohol consumption in the long run, both in the general population and in high-risk populations (e.g., heavier drinkers or adolescents and young adults). Yet Federal and State excise taxes, as well as overall alcoholic beverage prices, have increased only rarely and, when adjusted for inflation, have declined significantly over the years, according to Drs. Xin Xu and Frank J. Chaloupka. The authors review numerous studies exploring the relationship between alcoholic beverage taxes and alcohol consumption and its related consequences. (pp. 236–245)

## THE ALCOHOL POLICY INFORMATION SYSTEM (APIS) AND POLICY RESEARCH AT NIAAA

Measuring the effects of specific policies on alcohol-related behaviors and health outcomes presents a variety of daunting challenges. This sidebar by Mr. Greg Bloss summarizes how the Alcohol Policy Information System (APIS) was developed as a tool to support research on the effects and effectiveness of alcohol-related public policies. (pp. 246–247)

## REGULATING AVAILABILITY: HOW ACCESS TO ALCOHOL AFFECTS DRINKING AND PROBLEMS IN YOUTH AND ADULTS

Raising the minimum legal drinking age, having greater monopoly controls over alcohol sales, lowering outlet numbers and reducing outlet densities, and limiting hours and days of sale can effectively reduce alcohol sales, use, and problems. Dr. Paul J. Gruenewald briefly outlines the results of the work in these four areas, focusing on the minimum legal drinking age, the privatization of alcohol control systems, outlet densities, and hours and days of sale. (pp. 248–256)

## THE ROAD TO A WORLD HEALTH ORGANIZATION GLOBAL STRATEGY FOR REDUCING THE HARMFUL USE OF ALCOHOL

According to the World Health Organization (WHO), harmful alcohol use is a global problem that needs to be addressed on an international level. Dr. Maristela G. Monteiro discusses the WHO’s alcohol-related activities, including a global strategy to prevent and reduce the effects of harmful alcohol consumption. (pp. 257–260)

